# Novel application of amino-acid buffered solution for neuroprotection against ischemia/reperfusion injury

**DOI:** 10.1371/journal.pone.0221039

**Published:** 2019-09-10

**Authors:** Jiun Hsu, Chih-Hsien Wang, Shu-Chien Huang, Yung-Wei Chen, Shengpin Yu, Juey-Jen Hwang, Jou-Wei Lin, Ming-Chieh Ma, Yih-Sharng Chen

**Affiliations:** 1 Department of Cardiovascular Surgery, National Taiwan University Hospital Yunlin Branch, Yunlin, Taiwan; 2 Department of Cardiovascular Surgery, National Taiwan University Hospital and National Taiwan University College of Medicine, Taipei, Taiwan; 3 Department of Cardiovascular Medicine, National Taiwan University Hospital Yunlin Branch, Yunlin, Taiwan; 4 School of Medicine, Fu Jen Catholic University, New Taipei, Taiwan; University of PECS Medical School, HUNGARY

## Abstract

Ischemic neuron loss contributes to brain dysfunction in patients with cardiac arrest (CA). Histidine–tryptophan–ketoglutarate (HTK) solution is a preservative used during organ transplantation. We tested the potential of HTK to protect neurons from severe hypoxia (SH) following CA. We isolated rat primary cortical neurons and induced SH with or without HTK. Changes in caspase-3, hypoxia-inducible factor 1-alpha (HIF-1α), and nicotinamide adenine dinucleotide phosphate oxidase-4 (NOX4) expression were evaluated at different time points up to 72 h. Using a rat asphyxia model, we induced CA-mediated brain damage and then completed resuscitation. HTK or sterile saline was administered into the left carotid artery. Neurological deficit scoring and mortality were evaluated for 3 days. Then the rats were sacrificed for evaluation of NOX4 and H_2_O_2_ levels in blood and brain. In the *in vitro* study, HTK attenuated SH- and H_2_O_2_-mediated cytotoxicity in a volume- and time-dependent manner, associated with persistent HIF-1α expression and reductions in procaspase-3 activation and NOX4 expression. The inhibition of HIF-1α abrogated HTK’s effect on NOX4. In the *in vivo* study, neurological scores were significantly improved by HTK. H_2_O_2_ level, NOX4 activity, and NOX4 gene expression were all decreased in the brain specimens of HTK-treated rats. Our results suggest that HTK acts as an effective neuroprotective solution by maintaining elevated HIF-1α level, which was associated with inhibited procaspase-3 activation and decreased NOX4 expression.

## Introduction

In previous studies, we proved that extracorporeal cardiopulmonary resuscitation could improve survival rates and neurological outcomes compared with conventional cardiopulmonary resuscitation (CPR) [1,2]. However, brain damage caused by hypoperfusion and reperfusion injuries remains the leading cause of death among patients who survive cardiac arrest (CA) and intensive care was withdrawn for 30.9% of CA survivors because of poor neurological outcomes [3]. This finding was compatible with our own experience. In our cardiac surgery practice, we use a cardioprotective solution to protect patients’ hearts when we arrest them. This practice led us to wonder about potential neuroprotective solutions to protect the brain from damages by CA. Although many methods had been used to protect brain during CA that is induced for aortic arch surgery, there is no robust evidence to support available pharmacologic protections [4].

Histidine–tryptophan–ketoglutarate (HTK) solution was developed since the 1960s as a myocardial protecting solution [5]. HTK is a low-viscosity fluid with an electrolyte consistency similar to intracellular fluid. Histidine has a large buffering capacity that helps prevent the development of acidosis during the ischemic period. Tryptophan makes the cell membrane more stable, and ketoglutarate is a substrate in the Krebs cycle that facilitates high-energy phosphate formation during ischemia while also inhibiting glycolysis. HTK solution provides excellent and prolonged protection in hypothermic conditions and is now used increasingly for intra-abdominal organ preservation [6]. However, there are no reports on the use of HTK for brain protection during ischemia. Due to these beneficial characteristics of HTK, we considered that it might help protect the central nervous system from periods of ischemia and reperfusion injuries.

Hypoxia-inducible factor 1 (HIF-1) was identified in 1992 as a factor that responds within hypoxic environments [7], and its effects on cells during stress conditions were studied extensively thereafter. HIF-1 becomes functional once the α and β subunits form a heterodimer [8]. The α subunit is constantly consumed under normoxic conditions via the following sequence: hydroxylation by prolyl hydroxylase, ubiquitination by the Von Hippel–Lindau (VHL) complex, and degradation by proteasomes. Under hypoxic or stress conditions, the α subunit enters the nucleus and together with the β subunit forms a complex to bind the hypoxia-response element (HRE) and activate target genes. The genes influenced by HIF-1 provide protective effects on cell survival while also facilitating apoptosis or apopnecrosis in different hypoxic environments. HIF-1 also can bind to the promoter of the caspase-3 gene [9], thereby increasing the expression of procaspase-3. However, its relationship to increased apoptosis still requires clarification. When faced with a hypoxic environment, cells will try to maintain adequate amounts of superoxide by increasing the number of enzymes that produce it, and nicotinamide adenine dinucleotide phosphate oxidase 4 (NOX4) is the main connection to this process [10]. HIF-1 increases the expression of NOX4 by binding to HRE at the gene promoter region [11]. A gene knockout study that focused on ischemia/reperfusion injury of heart cells also showed decreased levels of HIF-1 related to the decreased nicotinamide adenine dinucleotide phosphate oxidase activity [12].

We tried to validate the hypothesis that HTK solution has neuroprotective capabilities during ischemic periods in the first part of this project, using an *in vitro* experiment of neural cells harvested from embryonic rats. The second part consisted of an *in vivo* study using the rat asphyxial CA (aCA) model [13], simulating CA scenario encountered clinically. The effects of HTK administration during aCA were evaluated.

## Materials and methods

### Primary culture of rat cortical neurons

Cortical neurons were isolated from embryonic Wistar rats (embryonic days 16 and 17), as described previously [14]. All animal treatments were approved by the Animal Care Committee of Fu Jen Catholic University (permit numbers: A10362) and all efforts were made to minimize suffering. Rats (BioLasco, Taipei, Taiwan) were housed in filter top cages with 1–2 rats per cage with specific-pathogen-free condition and cages were enriched with hardwood chips. In brief, the embryonic rats were removed from anesthetized dam (using halothane), and immediately rinsed in a neurobasal medium. The dam was then sacrificed by intravenous injection of sodium pentobarbital (150 mg/kg). The embryonic brain cortex was carefully dissected out, soaked in 0.1 m phosphate-buffered saline (PBS, pH 7.4) containing 0.6% glucose, collected by centrifugation, treated with trypsin for 15 min at 37°C and neutralized with trypsin inhibitor for 5 min. A cell suspension was prepared by repeated passage through a pipette and filtration through an 80-μm nylon mesh. This suspension was then diluted with neurobasal medium containing B-27, 0.5 mM glutamine (Merck, Darmstadt, Germany), 25 μM glutamate (Merck), 100 IU/mL penicillin, and 100 mg/mL streptomycin. The cells were counted by staining with trypan blue, diluted to a density of 10^6^ cells/mL in the same medium, and plated onto poly-d-lysine–precoated dishes. The cells were cultured at 37°C in 21% O_2_ and 5% CO_2_ in a humidified incubator (NU-5700, NuAire, Plymouth, MN, USA) for 12 days *in vitro* (DIV) to mature. All chemicals and culture medium were obtained from Thermo Fisher Scientific (Waltham, MA, USA) unless otherwise indicated. HTK solution, with the commercial name Custodiol [15], was obtained from Dr. Franz Köhler, Chemie GmbH (Bensheim, Germany).

### Induction of severe hypoxia (SH)

After DIV 12, the cultured neurons were divided into 2 groups. Before exposure to SH, the culture medium of SH groups were replaced with a mixture of neurobasal medium and HTK in various fractions (1/8, 1/4, and 1/2) of the total volume. Then the cultures were exposed to 2% O_2_ and 5% CO_2_ at 37°C in an O_2_-controlled incubator (NU-5731, NuAire, Plymouth, MN, USA) for up to 72 h. The responses of lactate dehydrogenase (LDH) and HIF-1α both confirmed SH [16].

### LDH levels and cell viability assays

LDH levels were measured in the cell culture medium (20 μL) of 2 separate dishes at time points of 0, 1, 2, 4, 8, 24, 48, and 72 h using a commercial kit (Roche Applied Science, Indianapolis, IN, USA), as described previously [17]. We used the 3-(4,5-dimethylthiazol-2-yl)-2,5-diphenyltetrazolium bromide (MTT, Sigma-Aldrich, St. Louis. MO, USA) assay to measure cell viability. Briefly, after culture medium removed, the cells were washed 3 times with PBS (pH 7.4). MTT (5 mg /mL in PBS) was then added to the cells for 4 h at 37°C. MTT formazan crystals were then dissolved in dimethyl sulfoxide (DMSO) and the optical density (O.D.) measured using an ELISA plate reader (Amersham-Pharmacia Biotech, Piscataway, NJ, USA) at 570 nm. Cell viability (%) was calculated using the following formula: (viable cells) % = (O.D. of treated sample/O.D. of control sample) × 100.

### Exogenous H_2_O_2_ treatment in cortical neurons

We exogenously administered H_2_O_2_ to the neurons to mimic oxidative stress caused by SH. H_2_O_2_ was added directly to the culture medium at concentrations of 100, 400, and 800 μM. Cell viability and LDH release were evaluated at the time points previously mentioned.

### Preparation of cell-free system for luminol-enhanced chemiluminescence (CL) determination

To confirm the scavenge effect of HTK solution on H_2_O_2_ is mediated by cell system. This study further tested whether HTK solution scavenges H_2_O_2_ in a cell-free system. H_2_O_2_ at concentrations of 0, 1, 10 μM were prepared in phosphate-buffered saline (PBS, pH 7.4) and immediately wrapped in aluminum foil and kept on ice until CL measurement, usually done within 2 h [18]. H_2_O_2_ solution in a volume of 0.2 mL was placed in a completely dark chamber of the ultrasensitive CL analyzing system (CLD-110, Tohoku Electronic Industrial Co., Sendai, Japan). After 50 s background level determination, 1.0 mL of 0.1 mM luminol (Sigma-Aldrich, St. Louis. MO, USA) in PBS (pH 7.4) was injected into the H_2_O_2_ test solutions. The CL was continuously monitored for an additional 200 s. After 200 s of recording, 0.2 mL of PBS, HTK, or recombinant bovine catalase (500 mU, Sigma-Aldrich) was then injected into the mixed solution to test their individual effect on H_2_O_2_ for 100 s of recording. The total amount of CL after 200 s of recording was calculated by integrating the area under the curve (AUC) and subtracting it from the background level. The assay was performed in duplicate for each test and was expressed as CL counts × 10 s after buffer or solution treatment.

### Induction of rat brain ischemic damage by asphyxia

For the *in vivo* portion of this study, we induced asphyxia in a rat model, as described previously [19]. All rats were adult male Wistar (BioLasco) aged between 11 and 12 weeks at the time of surgery. Briefly, rats were anesthetized with sodium pentobarbital (50 mg/kg intraperitoneally) and intubated. The left carotid artery was cannulated for antegrade solution delivery and the left femoral artery was cannulated for the recording of blood pressure and heart rate. The endotracheal tube was clamped to induce asphyxia for 4 min 30 s (the 4′30″ group) or 6 min 30 s (the 6′30″ group). We slowly infused 200 μL of saline or HTK (randomly allocated) via the left carotid artery catheter within the first minute of asphyxia. After induction of aCA, standard external CPR and intravenous epinephrine injections were carried out. Controlled ventilation using pure O_2_ was extended for 60 min after return of spontaneous circulation (ROSC). After extubation and wound closure, the rats were placed in plastic cages and observed continuously for 60 min and then intermittently for another 3 days. The first day after the procedure, rats received analgesia (5 mg/kg Carprofen, Sigma-Aldrich) twice a day via a subcutaneous injection. During this period, a heating blanket was placed under the cage for appropriate environmental control and food and water was placed inside the cage to promote accessibility. In addition, rats were weighted and were checked daily for signs of severe illness to determine whether early humane endpoints were reached. Criteria included weight loss of >15% in the first day, weight loss >20% if the rats were survived. A total of 40 male rats were used in the current study. All experiments are reported in accordance with the Animal research reporting of in vivo experiments (ARRIVE) guidelines. A neurological deficit (ND) evaluation was performed on the day of asphyxia after extubation (day 0) and after asphyxia on days 1, 2, and 3. After 3 days, all animals were humanely killed using an overdose of sodium pentobarbital (150mg/kg, intraperitoneally). The blood was sampled and the brain perfused transcardially with 0.1 M PBS (pH 7.4). We prepared the brains for determination of H_2_O_2_ and NOX4 mRNA levels from the brain tissue as in supplementary S1 Fig.

### Neurological deficit score

The ND scoring system is presented in Table 1 and has been described previously [20]. ND scores were obtained from 2 independent observers, with a third observer serving as an arbiter if there were any discrepancies.

**Table 1:** Neurological deficit (ND) scoring system in rat asphyxia.

		Score
Level of consciousness	Attempt to explore spontaneously	2
	No attempt to explore spontaneously	0
Corneal reflex	Present	2
	Absent	0
Respiration	Normal	2
	Abnormal	0
Righting reflex (attempting to right self when placed on back)	Present	2
	Absent	0
Coordination	Normal	2
	Worst	0
Movement activity (legs/tail movement)	Normal	2
	Stiff	1
	Paralyzed	0

Total score: 12 = normal, 0 = brain dead.

### Quantitation of H_2_O_2_ in culture medium and brain cortex

H_2_O_2_ was measured using Amplex red (Molecular Probes, Eugene, OR, USA) [21]. Briefly, 200 μM of Amplex red and 1 U/mL of horseradish peroxidase were added to 20 μL of culture medium, tissue homogenates of brain cortex, or a standard H_2_O_2_ solution in PBS (pH 7.4). The samples were incubated for 30 min in 96-well microplates in the dark and at room temperature. Fluorescence intensity was measured using an automatic microplate reader (model KC4, Bio-Tek Instruments, Winooski, VT, USA) at an excitation wavelength of 530 nm and an emission wavelength of 590 nm. After subtracting background fluorescence, H_2_O_2_ concentrations in the culture medium were calculated on the basis of an H_2_O_2_ standard curve.

### Western blot analysis for protein expression

The expressions of full-length (total) caspase 3, cleaved caspase 3, NOX2 and NOX4, HIF-1α, and actin were examined by immunoblot analysis in the cultured neurons and brain tissue, as described previously [17]. Primary antibodies against the above proteins were obtained from Santa Cruz Biotechnology (Santa Cruz, CA, USA). Briefly, equal amounts of cytosolic protein were separated in denaturing SDS polyacrylamide gels and electrophoretically transferred to polyvinylidene difluoride membranes (Amersham, Little Chalfont, UK). The membranes were then incubated with the appropriate primary antibody (sc-1225, 1:1000 for caspase 3; sc-5827, 1:1000 for NOX2; sc-21860, 1:2500 for NOX4; sc-10790, 1:2000 for HIF-1α; sc-8432, 1:1000 for actin) overnight at 4°C. After washing, the membranes were incubated for 1 h at room temperature with the corresponding horseradish peroxidase–conjugated secondary antibodies (1: 200; Vector Laboratories, Burlingame, CA, USA). Bound antibodies were visualized using an enhanced chemiluminescence kit (Amersham-Pharmacia Biotech) and Kodak film. Band density was measured semiquantitatively using an image analysis system (Diagnostic Instruments, Sterling Heights, MI, USA). The amount of each protein was expressed relative to the amount of actin.

### Measurement of NOX activity in brain tissue

NADH (0.1 mmol/L) was used as a substrate for NOX activity assay, as described previously [22]. Salmon testis DNA was added to the reaction mixture to stabilize ethidium fluorescence and increase the sensitivity of the assay. Enzyme activity is presented as fluorescence units/10 s/mg protein.

### Quantitative real-time PCR for NOX4 expression

RNA was extracted using a commercial kit (RareRNA, Bio-East Technology, Taipei, Taiwan), as previously described [23], and cDNA was synthesized at 42°C for 45 min (reaction mixture: 2 μg of RNA, 5 μg of poly(dT)15 oligonucleotide primer (Life Technologies, Waltham, MA, USA) and exposed to 200 units of reverse transcriptase (Moloney murine leukemia virus; Promega, Madison, WI, USA). Quantitative RT-PCR was performed in an ABI StepOne Plus system (Applied Biosystems, Foster City, CA, USA). PCR was performed using 100 ng of cDNA and 30 μmol of primers (total reaction volume, 20 μL) using the SYBR Green PCR master mix kit, according to the manufacturer’s instructions (Applied Biosystems). The following primers were used for PCR: NOX4: 5′-GGA TCA CAG AAG GTC CCT AGC-3′ (forward) and 5′-AGA AGT TCA GGG CGT TCA CC-3′ (reverse); glyceraldehyde 3-phosphate dehydrogenase (GAPDH): 5′-TTA GCA CCC CTG GCC AAG G-3′ (forward) and 5′-CTT ACT CCT TGG AGG CCA TG-3′ (reverse). The cycling conditions were as follows: 95°C for 20 s, followed by 40 cycles of 95°C for 1 s and 60°C for 20 s. A melting curve analysis was performed at the end of each PCR experiment. All reactions were run in duplicate. The ΔCt (threshold cycle) was calculated by subtracting the raw Ct values for GAPDH from the raw Ct values for the target gene, thereby providing information about relative changes in gene expression. Changes in NOX4 expression were calculated as 2^−ΔCt^ and expressed as fold change relative to that of the 4′30′′ group.

### Statistical analysis

All data are expressed as mean ± standard error of the mean (SEM). Statistical analysis was performed using analysis of variance, or analysis of variance followed by the Newman–Keuls method for multiple comparisons between groups. Kaplan–Meier method with log-rank test was used in the survival analysis of animal study. A *p* value < 0.05 was considered significant.

## Results

**Fig 1:**
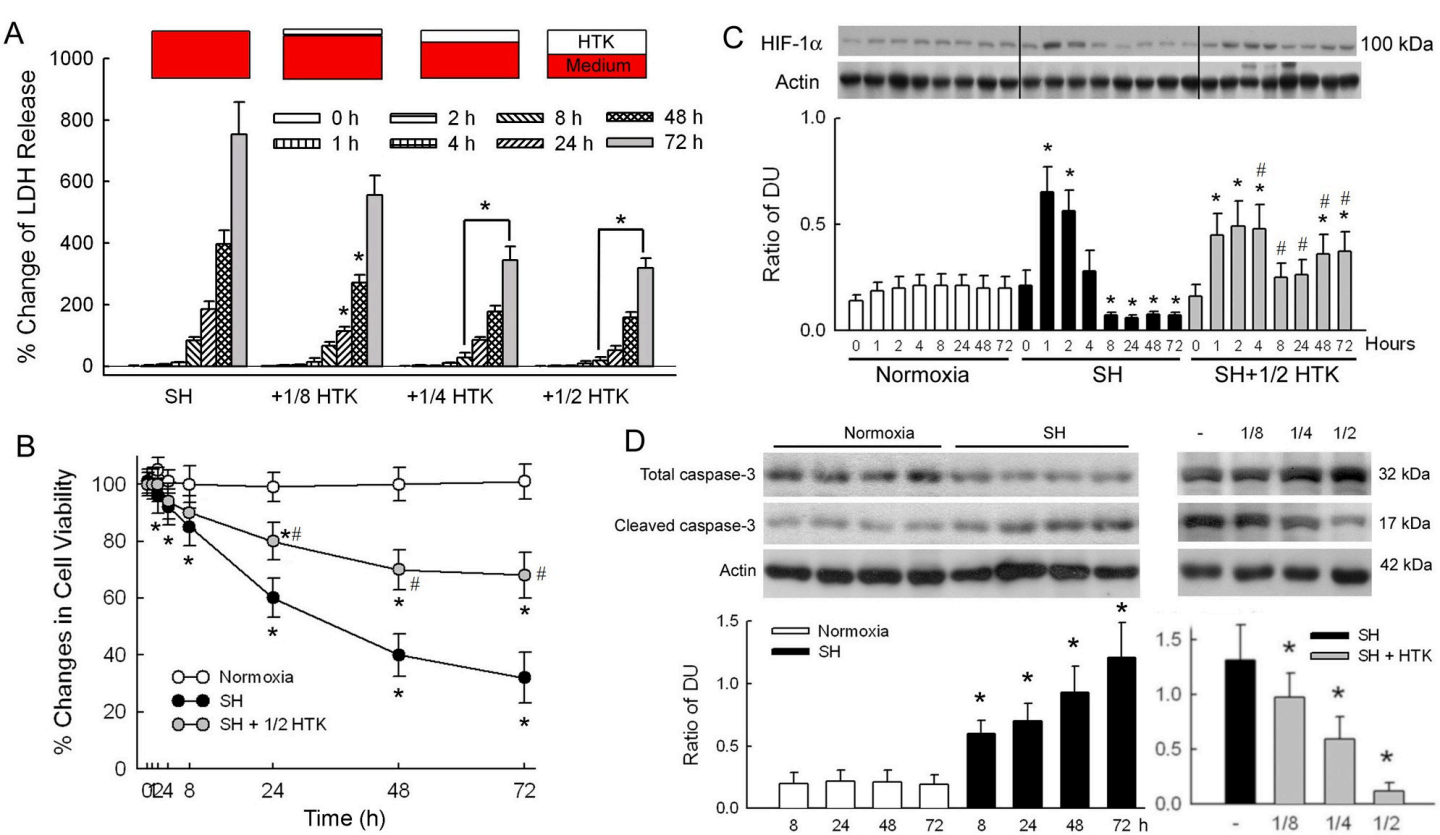
Responses of cultured rat cortical neurons during severe hypoxia (SH). Using different ratios of histidine–tryptophan–ketoglutarate (HTK) solution in volume, the response of cultured rat cortical neurons during SH was evaluated. (A) Lactate dehydrogenase (LDH) released under SH. (B) Cell viability under normoxia, SH and SH + 1/2 HTK, was evaluated using 3-(4,5-dimethylthiazol-2-yl)-2,5-diphenyltetrazolium bromide (MTT) assay. (C) Hypoxia-induced factor-1α (HIF-1 α) expression under normoxia, SH, and SH + 1/2 HTK. (D) The ratio of cleaved caspase-3 to total caspase-3 under normoxia and SH (left bar graph). The same ratio with different proportion of HTK. (*N* = 6 experiments performed at each time point. * *p* < 0.05 as compared with corresponding controls at the same time points as normoxia in A and C, right bar graph of D, and SH in B, and *p* < 0.05 in left bar graph of D compared with the untreated SH group at 72 h, # *p* < 0.05 as compared with normoxia in B and SH in C at the same time points).

HTK solution was used for cardioprotective solution during cardiac surgery for decades and is now also an organ preservative solution in the transplantation of intra-abdominal organs. The hypothesis of using HTK to protect brain during total circulatory arrest period of cardiac surgery or cardiac arrest (CA) was raised and we tested this hypothesis using cellular and animal models. Our results showed that during SH, the percentage change of LDH increased significantly after 8 h, as shown in Fig 1A. Using 1/8 HTK solution in the culture medium, the percentage change of LDH decreased significantly at 24 and 48 h compared with the SH group at the same time points (*p* < 0.05). The protective effect becomes more obvious and extended to 72 h if the proportion of HTK solution increased to 1/4 or 1/2. Cell viability was well preserved in the normoxic group, as in Fig 1B, and decreased gradually to 32% at 72 h in the SH group. Substituting half the culture medium with HTK solution significantly increased cell viability compared with the SH group at 24, 48, and 72 h (*p* < 0.05). A control group of HTK usage under normoxic environment was also performed, and the results show that long-term exposure (72 h) of neurons to HTK of any proportion is harmful, as in supplementary S2 Fig.

Within the normoxia group, HIF-1α maintained a stable level during the observation period, as seen in Fig 1C. Exposing the cell cultures to SH increased HIF-1α levels dramatically at 1 and 2 h (*p* < 0.05 compared to the normoxia group at the same time points). HIF-1α then decreased after 8 h to levels below those of the normoxia group. Regarding SH+1/2 HTK, HIF-1α increased significantly after ischemia. Although there was a decrease at 8 and 24 h, the HIF-1α levels were still higher than those of the normoxia group. HIF-1α levels increased and remained high at 48 and 72 h (*p* < 0.05 compared to normoxia and SH groups at the same time points).

The ratio of cleaved caspase-3 to total caspase-3 remained low during the study period for the normoxia group, as shown in Fig 1D. But it increased with the advance of the hypoxic period for the SH group. This ratio at 72 h decreased significantly even when the culture medium consisted of only 1/8 HTK, and became more obvious as proportion of HTK increased (*p* < 0.05 compared to the SH group).

**Fig 2:**
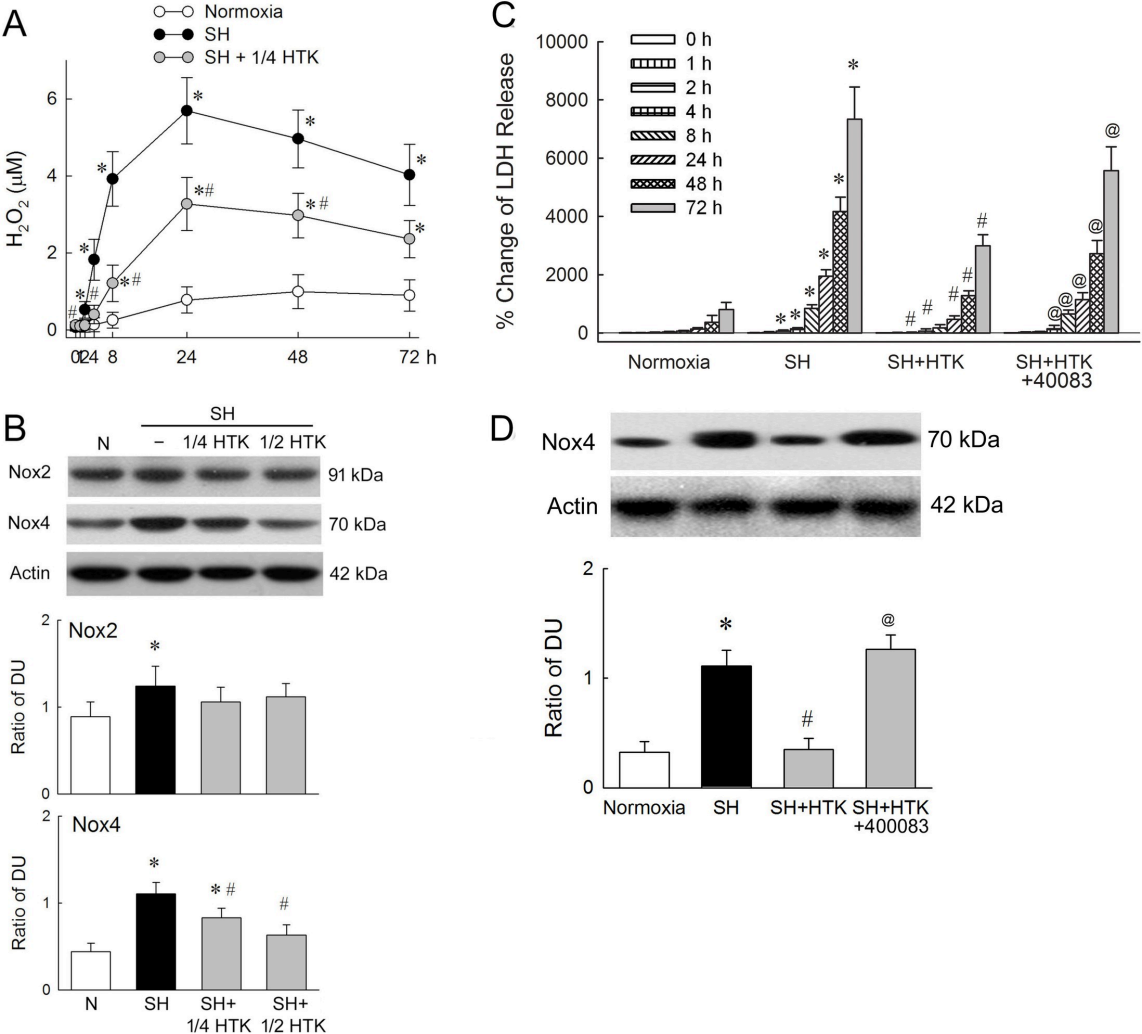
The association of nicotinamide adenine dinucleotide phosphate oxidase (NOX) and hypoxia-induced factor 1α (HIF-1α) during severe hypoxia (SH). Concentration of H_2_O_2_ release, NOX2 and NOX4 measured during the severe hypoxia (SH) period, with the association hypoxia-induced factor 1α (HIF-1α). (A) The concentration of H_2_O_2_ released under normoxia, SH and SH + 1/4 HTK (histidine–tryptophan–ketoglutarate). (B) At 72 h, NOX2 and NOX4 expression under normoxia, SH, SH + HTK. (C) Release of lactate dehydrogenase (LDH) in response with HIF-1 antagonist 400083. (D) Expression of NOX4 at 72 h of SH, and the influence of HTK and HIF-1 antagonist 400083. (*N* = 6 experiments performed at each time point. * *p* < 0.05 compared to normoxia control, # *p* < 0.05 compared to SH group, @ *p* < 0.05 compared to SH + HTK group).

During the observation period under the normoxia condition, the concentration of H_2_O_2_ increased slightly, but exposure to SH peaked H_2_O_2_ concentration at 24 h, remaining high until 72 h (*p* < 0.05 compared to the normoxia control); the use of 1/4 HTK did not prevent H_2_O_2_ production, but the level was significantly lower than in the SH group throughout the whole observation period (*p* < 0.05). The levels of NOX2 and NOX4 measured at 72 h (Fig 2B). The expression of NOX2 was not influenced by the presence of HTK during SH, but NOX4 expression decreased significantly as we titrated up the proportion of HTK during SH (*p* < 0.05 compared to the SH group). As shown in Fig 2C, using an HIF-1 antagonist (400083) on the SH group with HTK, the percentage change of LDH release were significantly increased from 4 h to 72 h as compared to the SH group with HTK (*p* < 0.05); however, the levels remained lower than those observed in the SH group. The level of NOX4 expression at 72 h (Fig 2D) decreased after using HTK (*p* < 0.05 as compared to SH group), but returned to levels similar to those of the SH group if the HIF-1α antagonist was added (*p* < 0.05 as compared to SH group with HTK). HIF-1 decreases NOX4 levels under the influence of HTK, as indicated by our results.

**Fig 3:**
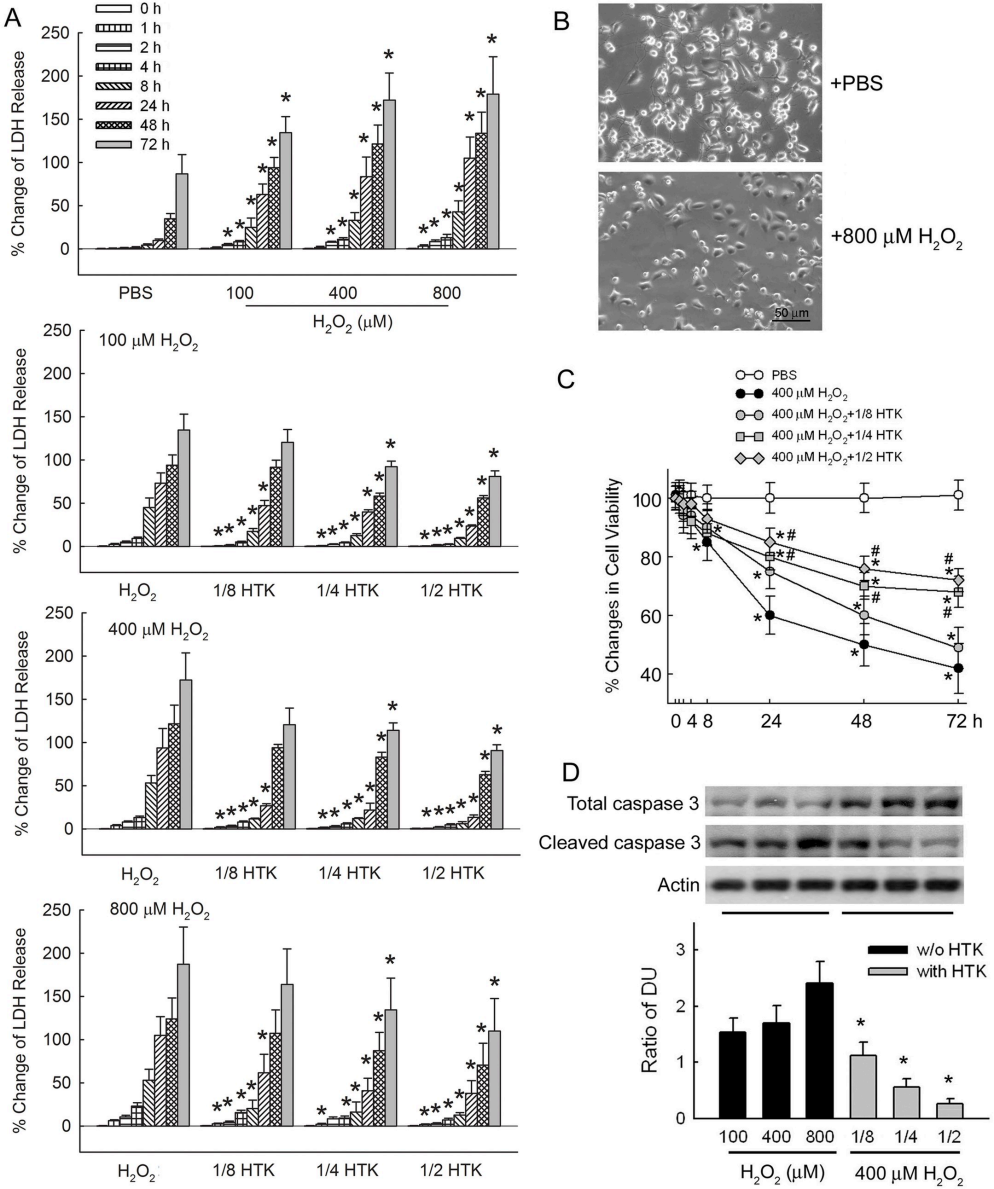
H_2_O_2_ administration to rat cortical neuron cultures, mimicking oxidative stress during severe hypoxia (SH). (A) Lactate dehydrogenase (LDH) release using different H_2_O_2_ concentration. (*p < 0.05 compared to phosphate-buffered saline (PBS) control). For each H_2_O_2_ concentration, LDH release under the effect of histidine–tryptophan–ketoglutarate solution (HTK) (*p < 0.05 compared to the same H_2_O_2_ group without HTK). (B) A small amount of viable cells under 800 μM H_2_O_2_. (C) The PBS + 400 μM H_2_O_2_ group was used for cell viability test. (*p < 0.05 when compared to the PBS control, and #*p* < 0.05 when comparing 1/2 or 1/4 HTK with no HTK). (D) The ratio of cleaved caspase-3 to total caspase-3 after H_2_O_2_ challenge and HTK effect. (*p < 0.05 compared to 400 μM H_2_O_2_ without HTK). (*N* = 6 experiments performed at each time point).

As shown in Fig 3A, challenges with different H_2_O_2_ concentrations resulted in significant increases in the percentage change of LDH release after 2 h, and even only 1 h after challenged with 800 μM H_2_O_2_ (*p* < 0.05 compared to PBS control at each time point). When HTK was present, neuronal cells became more resistant to H_2_O_2_ damage. Adding 1/8 HTK decrease the percentage change of LDH release significantly (*p* < 0.05) in all groups exposed to different concentration of H_2_O_2_ from 1 to 24 h. The protective effect extended to 72 h in the different concentrations of H_2_O_2_ + 1/2 HTK groups (*p* < 0.05).

Cell viability was evaluated in the PBS group without H_2_O_2_ and various concentrations of HTK challenged with 400 μM H_2_O_2_. Small amount of viable cells remained in the + 800 μM H_2_O_2_ group as in Fig 3B. The + 400 μM H_2_O_2_ group significantly attenuated cell viability at all time points. The cell viability of the group co-treated with 1/8 HTK was significantly better than the + 400 μM H_2_O_2_ group only over the first 24 h (*p* < 0.05). On the other hand, the 1/4 and 1/2 HTK groups showed significantly better cell viability through 72 h of the observation period (*p* < 0.05 as compared to the + 400 μM H_2_O_2_ group).

The ratio of cleaved caspase-3 to total caspase-3 indicated caspase-3 activation, which increased as the concentration of H_2_O_2_ was increased. As the proportion of HTK increased from 1/8 to 1/2 of the culture medium, caspase-3 activation decreased significantly when challenged with 400 μM H_2_O_2_ (*p* < 0.05 compared to the PBS + 400 μM H_2_O_2_ group, as shown in Fig 3D).

**Fig 4:**
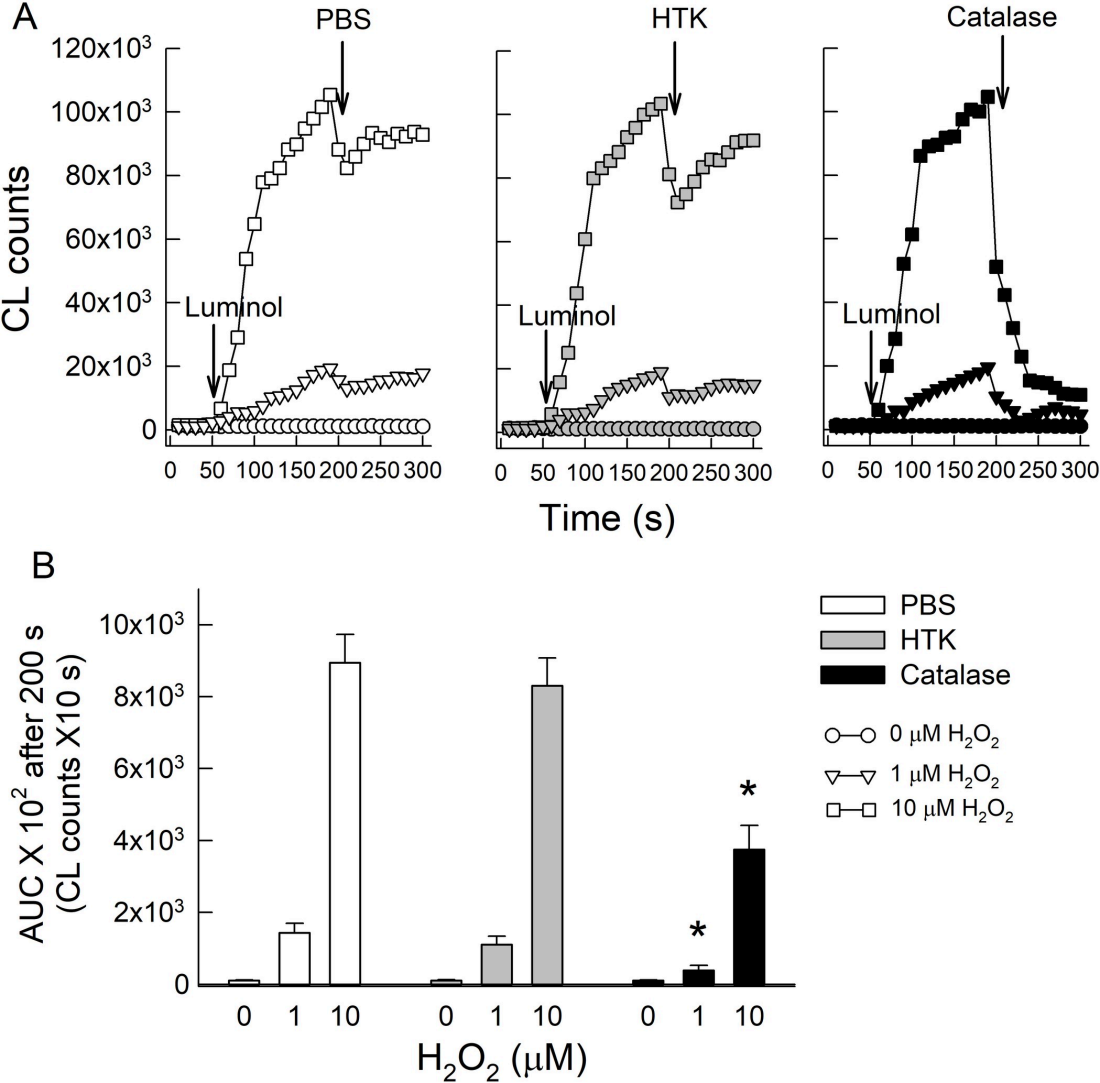
Effects of HTK on H_2_O_2_ scavenge in a neuron-free system. (A) The representative recordings show luminol-mediated CL counts were dose-dependently increased in response to different concentrations of H_2_O_2_ after 50 s of background (the first arrow). The PBS, HTK, or recombinant catalase was then treated to examine their antioxidant effects (the second arrow at 200 s). PBS and HTK show similar changes in CL count as first drop and then return (the left and middle panels), and only catalase decreases CL count continuously (the right panel). (B) The area under curve (AUC) after 200 s of recording was calculated and showed that there is no significant difference between PBS or HTK, and only catalase significantly lowers CL counts. Data are expressed as means ± SEMs (n = 6). *P < 0.05 vs. PBS at the same concentration of H_2_O_2_.

Together with the cell study, our results showed that HTK mediates cellular activation of HIF-1α against H_2_O_2_-induced neuronal injury in SH. We further tested if the effect of HTK is dependent on cellular system or antioxidant nature of HTK in terms of neuroprotection. The H_2_O_2_ scavenger ability of HTK was evaluated in a cell-free system by monitoring changes in luminal-based chemiluminescence (CL) counts. The representative recordings shown in Fig 4A indicate an initial drop in CL counts while PBS or HTK was added (left and middle panels) because of dilution effect, but then increased to a stable state shortly after. The recombinant catalase added (right panel) could validate that the increase in CL counts after injection of luminol was due to the presence of H_2_O_2_. The total amount of CL after 200 s of recording in Fig 4B did not show a significant difference between the PBS and HTK solution on H_2_O_2_ scavenging activity. Catalase, however, significantly attenuated the total CL amounts induced by H_2_O_2_.

**Fig 5:**
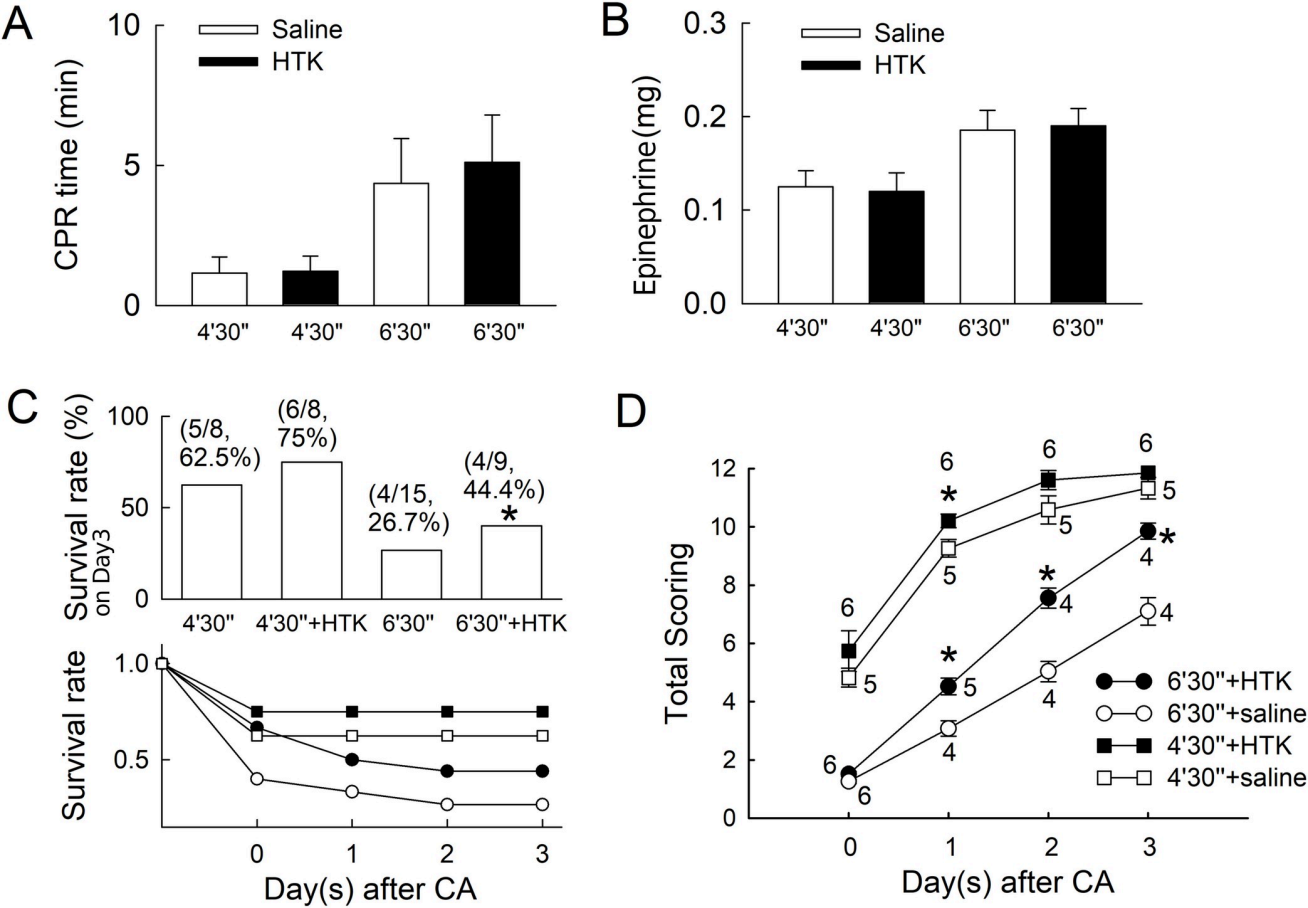
Results of cardiopulmonary resuscitation (CPR) for asphyxial cardiac arrest (aCA). The CPR time, epinephrine used during CPR, survival rate and neurological deficit (ND) scores in rats, were compared in saline and histidine–tryptophan–ketoglutarate solution (HTK) groups. (**A**) CPR time (*p* < 0.05 for the 6’30″ groups (endotracheal tube clamped and asphyxia for 6 min 30 s) compared to the 4’30″ groups (endotracheal tube clamped and asphyxia for 4 min 30s)). (**B**) The epinephrine dose used for CPR. (**C**) Survival rate on day 3. (*p < 0.05, HTK vs. saline in the 6′30″ group). (**D**) Total neurological deficit (ND) scores are shown from day 0 to day 3. Numbers listed beside each icon on the line chart are the survived and observed rats at that time point. Treatment with HTK improved ND scores significantly at day 1 for the 4′30″ group and from day 1 to day 3 for the 6′30″ group. (* *p* < 0.05 compared with the saline-treated controls with the same duration time of aCA).

Using a rat model, we induce CA after asphyxia by clamping endotracheal tube. The asphyxia time was measured from the clamping of the endotracheal tube to the start of resuscitation. Standard chest compression, endotracheal tube ventilation and epinephrine were applied to facilitate returning of spontaneous circulation. Epinephrine was used because it could increase arterial pressure and coronary perfusion pressure, and provide good successful resuscitation rate while low dosage was used [24,25]. The 4′30″ groups (asphyxia for 4 min and 30 s) had shorter durations of resuscitation, and the 6′30″ groups (asphyxia for 6 min and 30 s) were resuscitated significantly longer (*p* < 0.05 as compared to the 4′30″ groups with the same treatment), but the difference in resuscitation duration between the saline and HTK groups was not significant at the same asphyxia time (Fig 5A). For the epinephrine dosage, the 6′30″ groups used nearly twice the dosage as the 4′30″ groups. No significant differences were observed between the HTK group and the saline group with the same asphyxia time (Fig 5B).

The survival rate was significantly decreased when the asphyxia time increased from 4 min 30 s to 6 min 30 s (*p* < 0.05 when comparing saline and HTK subgroup with different asphyxia time). The use of HTK increased rat survival from 62.5% to 75% for the 4′30″ group without statistical significance (*p* = 0.6547), and significantly from 26.7% to 44.4% for the 6′30″ group at day 3 (*p* < 0.05, Fig 5C). The infarction size of the 4′30″ groups was lower than that of the 6′30″ groups, and was lower in the groups treated with HTK than that of saline-treated groups; however, these were not statistically significant. The total neurological deficit (ND) scores between the HTK and saline subgroups were similar among the 4′30″ groups. For the 6′30″ groups, the total ND score increased significantly for the HTK subgroup (*p* < 0.05) compared with the saline subgroup at day 1 and the gap increased gradually to day 3 (Fig 5D).

**Fig 6:**
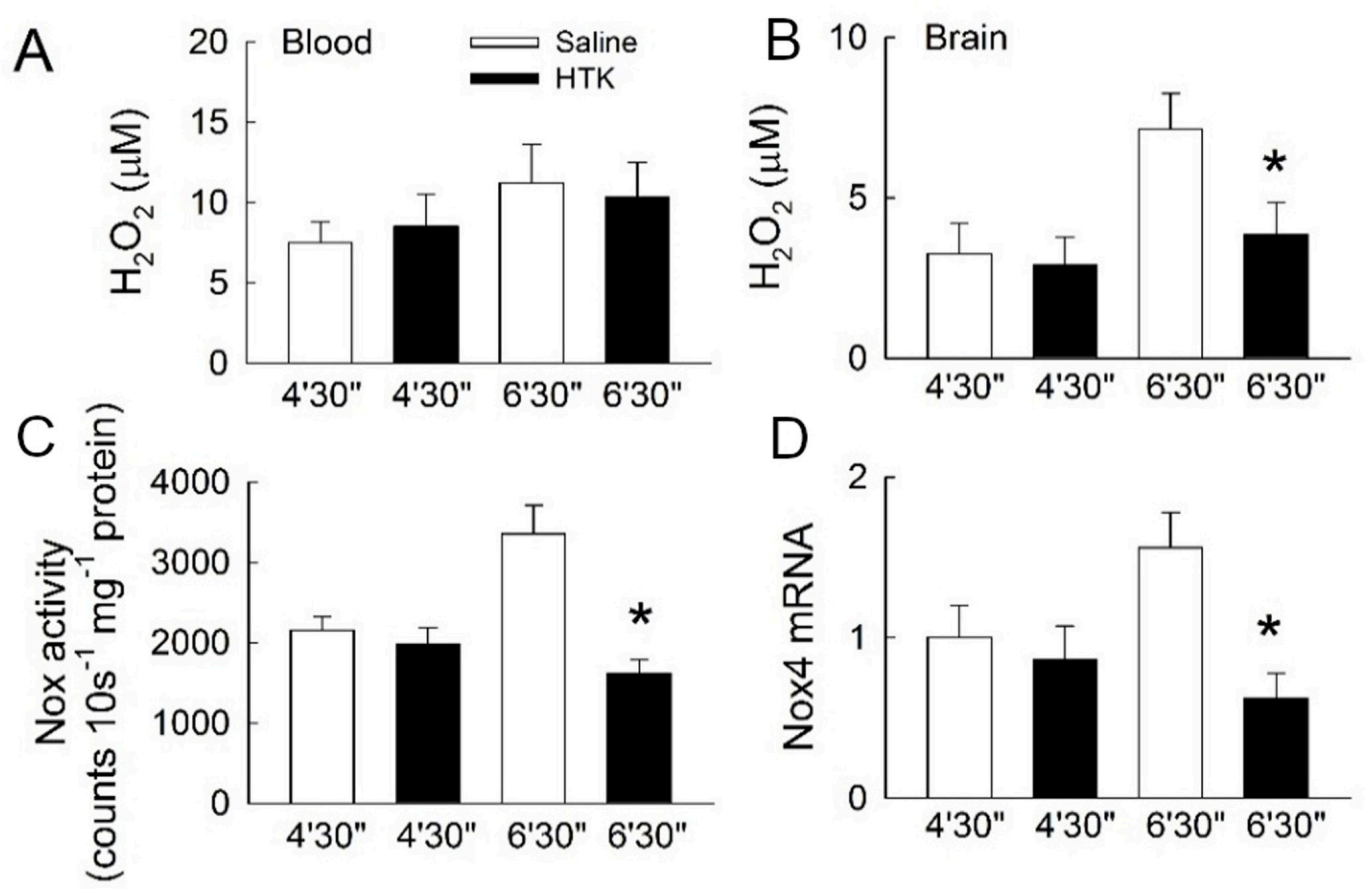
Effects of histidine–tryptophan–ketoglutarate solution (HTK) on H_2_O_2_ production and brain tissue nicotinamide adenine dinucleotide phosphate oxygenase 4 (NOX4) expression. Changes in H_2_O_2_ levels in blood (**A**) and brain cortex (**B**). Changes in NOX activity (**C**) and NOX4 mRNA expression (**D**) in rat brain cortex. (*N* = 5 experiments for 4′30″ + saline group, *N* = 6 for 4′30″ + HTK group, *N* = 4 for 6′30″ + saline and 6′30″ + HTK groups. * *p* < 0.05 compared with saline-treated controls with the same asphyxial time of CA).

The blood H_2_O_2_ concentrations at 72 h after return of spontaneous circulation (ROSC) were similar between the HTK and saline groups at each asphyxia time (Fig 6A), but the H_2_O_2_ concentration of brain cortex remained significantly higher 72 h after ROSC in the saline group compared with the HTK group (*p* < 0.05, Fig 6B). The expression of both NOX4 mRNA and NOX4 activity showed this similarity. High NOX4 mRNA expression, with increased NOX4 activity, could be observed in the saline group compared with the HTK group in the 6′30″ group (*p* < 0.05, Fig 6C and 6D).

## Discussion

Professor Bretschneider reported that if HTK is used as a cardioplegic agent, the adenosine triphosphate (ATP)-time (period until ATP content had dropped to 4 μmol/g wet weight) could be prolonged 8–9 times at 5°C [5]. Ketoglutarate was included in HTK solution because of its ability to enter Krebs’ cycle and provide ATP during ischemic environment. Glutamate was found to be a survival factor in an in vitro model of neuronal hypoxia/reoxygenation injury because it could be transformed into ketoglutarate in a hypoxic situation with the orchestration of sodium calcium exchanger (NCX) and excitatory amino-acid transporters [26]. In an isolated rat heart study, the ratio of ATP and adenosine diphosphate (ADP) could be maintained over 50% after 8 h of cold storage using HTK as a preservative [27]. The low calcium content of HTK also decreases the risk of cell death caused by excessive intracellular calcium resulting from loss of ion homeostasis in severe hypoxia (SH), and decreases the burden of plasma membrane calcium ATPase pump and NCX exit mode activity [28]. We encountered ischemic brain injury in patients rescued by CPR during our daily practice. We applied HTK solutions to the neuronal cells and brain in an animal model and tested it as a potential neuroprotective agent due to its success in other organs.

In our study of rat cortical neuronal cells, we demonstrated that the release of LDH, due to cell damage caused by either SH or H_2_O_2_, was significantly decreased when we increased the proportion of HTK in the culture medium. Using 1/2 HTK in the culture medium, cell survival increased dramatically to 68.5% (*p* < 0.05) compared to 32% without HTK after ischemia for 72 h. HTK also increased cell survival from 42% to 72% (*p* < 0.05) after exposure to 400 μM H_2_O_2_ for 72 h. This is the first report of HTK usage on *in vitro* neuronal cells with definite protective effect during hypoxic condition and oxidative stress.

Without HTK, HIF-1 increased sharply at 1 h after exposure to the hypoxic environment, but the level of HIF-1 started decreasing at 2 h and decreased dramatically at 8 h of persistent ischemia, even lower than the sham group. This surge of HIF-1 was well illustrated in previous studies showing that the degradation of HIF-1 would be hindered during hypoxia [8]. With the aid of HTK, this surge of HIF-1 did not seem so abrupt. Although it decreased from the peak at 8 and 24 h of SH, the level of HIF-1 remained significantly higher at the 48 h and 72 h time points. HTK seems to keep neuronal cells capable of maintaining a high level of HIF-1.

HIF-1 was accused of promoting cell death after ischemia, essentially the opposite of its effects in helping cell survival during periods of ischemia. HIF-1α and P53 may “conspire to promote a pathological sequence resulting in cell death,” according to Halterman et al. [29].

Another study, published by Hoecke et al. [9], connected HIF-1 to caspase-3 and proved that HIF-1 binds directly to the promoter of the caspase-3 gene, increasing the amount of caspase-3 after photothrombotic cerebral ischemia. The expressions of HIF-1α and procaspase-3 were high and co-localized in the penumbra region and so was the cleaved procaspase-3. Cleaved procaspase-3 dominated in the infarction core with very low HIF-1α expression. Together with our finding that persistent high level of HIF-1α resulted in decreased ratio of cleaved procaspase-3 to total caspase-3, HIF-1α might also participate in the process of inhibiting procaspase-3 activation besides enhancing procaspase-3 gene expression. Thus, the duality of HIF-1α is not confined to the activation of hypoxia adaptation genes and the interaction with p53 for apoptosis [30], but might also include increased expression of the caspase-3 gene and the inhibition of the procaspase-3 activation process.

HIF-1α also bind directly to NOX4 gene promoter, and was responsible for the increased expression of NOX4 [11]. That study showed high HIF-1α levels during the first 4 h of hypoxia, peaking expression of NOX4 mRNA at 4–8 h after hypoxia, and elevating H_2_O_2_ levels gradually until 24 h after the onset of hypoxia. The elevation of NOX4 expression was also observed 12–24 h after performing transient middle cerebral artery occlusion in another study [31]. Oxidative stress, blood–brain barrier leakage, and neuronal apoptosis all decreased if NOX4 was knocked out or if its function was blocked after the ischemic period. In our study, when the HIF-1α levels were persistently elevated under the influence of 1/2 HTK in culture medium, NOX4 was suppressed to the level of the control group. The protective effect of HTK on neuronal cells during hypoxia might also relate to suppressed NOX4 expression by persistently elevated HIF-1α. Because there was no significance between PBS and HTK in H_2_O_2_-mediated changes in CL counts in a cell-free system, this clearly indicates that the effect of HTK against NOX4-mediated oxidative stress is dependent on cellular signal transduction in triggering neuroprotection.

In the second part of this study, a rat model of asphyxial cardiac arrest was used. This model was chosen because of its consistency in inducing global hypoxic–ischemic brain insult [32], which was encountered in the clinical scenario of CA. Our results show that exposure to HTK improved the neurological scores of rats in the first 3 days after aCA. NOX4 had already been identified as an important factor relating to brain autotoxicity and blood–brain barrier disruption after temporary middle cerebral artery occlusion [33]. The NOX4 knockout rats in that study had decreased infarction size with improved neurological and motor functions. Similar functional improvement was observed in our study. The improvement of ND scores was more prominent in the HTK 6′30″ group compared to the saline-treated 6′30″ group. This result was associated with decreased NOX4 function and decreased H_2_O_2_ concentrations that occurred in brain tissue and blood at day 3. In brain tissue of the saline group, persistent elevation of NOX4 mRNA levels, NOX4 function, and H_2_O_2_ concentration at day 3 indicated that the ischemic insult might result in reperfusion injury that last several days. The infusion of HTK during the second part of this report showed a decreased NOX4 expression and activity, and decreased H_2_O_2_ concentration, which is compatible with our *in vitro* study. This suggests that HTK might be suitable for use during resuscitation to improve neurological outcomes.

The guidelines for resuscitation have evolved in recent years. Early defibrillation, continuous chest compressions with minimal interruption, early epinephrine injections, and transport to capable hospitals have gained wide acceptance [34], and the target temperature management for comatose patients after resuscitation was granted level I recommendation in 2015 [35]. However, pharmacologic agents to increase neurological recovery are not yet widely available. Erythropoietin and its derivatives were effective in reducing ischemic size in an isolated heart model [36] and an animal brain infarction model [37]. Hydrogen inhalation combined with target temperature management improved study animal survival and neurological recovery after resuscitation from ventricular fibrillation [38]. Despite these good results, few clinical trials were reported [39]. But HTK is already used clinically.

The limitation of this study were that we did not elucidate the connection between persistently elevated HIF-1 levels with procaspase-3 and NOX4, and that HTK was infused at the beginning of asphyxia instead of the start of CPR.

## Conclusions

In conclusion, HTK solution maintained high HIF-1α levels in a neurological cell ischemia study, associated with increased procaspase-3 production, suppressed procaspase-3 activation, and decreased NOX4 expression. HTK infusion during aCA increased short-term neurological scores in survived animals which was associated with decreased NOX4 expression. This is the first report of a possible brain-protective solution for patients undergoing resuscitation or when long-term brain ischemia is needed to perform cardiac surgery.

## Supporting information

S1 Fig Photographs illustrating the coronal sections of rat brain slices stained with TTC. For determining infarction size (IS) after asphyxial cardiac arrest (aCA), rat brains were sliced coronally for groups of 4 min 30 sec (4’30”) and 6 min 30 sec (6’30”), and saline-treated or histidine–tryptophan–ketoglutarate solution (HTK)-treated aCA rats as described in the text. Non-ischemic areas are colored red, whereas, ischemic areas are pale. Note the missing (empty) slices were applied to determine tissue contents of H_2_O_2_ and protein activity and mRNA expression of NADPH oxidase-4 (NOX4).
(TIF)

S2 Fig The response of cultured rat cortical neurons during normoxia using different ratios of HTK (histidine–tryptophan–ketoglutarate solution) in volume. Under normoxia, the culture medium was substituted by HTK with 1/8, 1/4 and 1/2 in volume similar to those neuronal cells exposed to hypoxia. The percentage change of LDH release increased significantly at 72 h, which indicated that PBS solution was better as a neuronal cell culture medium. (*N* = 6 experiments performed at each time point. * *p* < 0.05 as compared with corresponding controls at the same time points)
(TIF)

S1 File The raw data of this report. All raw data were listed in this file to support our results in all figures.
(PDF)
