# Supplementary figures and images for: Novel application of amino-acid buffered solution for neuroprotection against ischemia/reperfusion injury
Source: PLoS One. 2019 Sep 10;14(9):e0221039. doi: 10.1371/journal.pone.0221039 (PMC6736298; doi:10.1371/journal.pone.0221039)

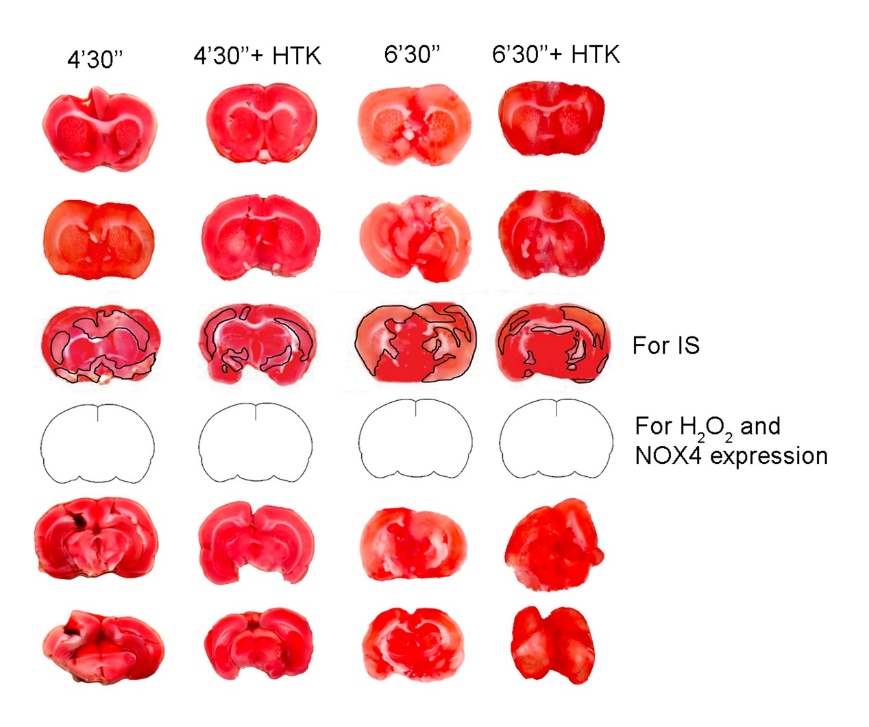

Supplement: S1 Fig — For determining infarction size (IS) after asphyxial cardiac arrest (aCA), rat brains were sliced coronally for groups of 4 min 30 sec (4’30”) and 6 min 30 sec (6’30”), and saline-treated or histidine–tryptophan–ketoglutarate solution (HTK)-treated aCA rats as described in the text. Non-ischemic areas are colored red, whereas, ischemic areas are pale. Note the missing (empty) slices were applied to determine tissue contents of H2O2 and protein activity and mRNA expression of NADPH oxidase-4 (NOX4). (TIF) [file pone.0221039.s001.tif]

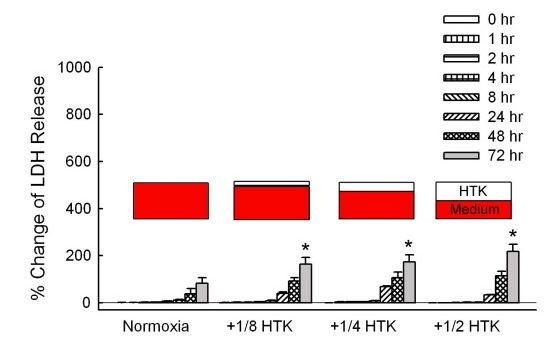

Supplement: S2 Fig — Under normoxia, the culture medium was substituted by HTK with 1/8, 1/4 and 1/2 in volume similar to those neuronal cells exposed to hypoxia. The percentage change of LDH release increased significantly at 72 h, which indicated that PBS solution was better as a neuronal cell culture medium. (N = 6 experiments performed at each time point. * p < 0.05 as compared with corresponding controls at the same time points) (TIF) [file pone.0221039.s002.tif]
