# Supplementary material for: Novel application of amino-acid buffered solution for neuroprotection against ischemia/reperfusion injury
Source: PLoS One. 2019 Sep 10;14(9):e0221039. doi: 10.1371/journal.pone.0221039 (PMC6736298; doi:10.1371/journal.pone.0221039)
Supplement: S1 File — All raw data were listed in this file to support our results in all figures. (PDF) [file pone.0221039.s003.pdf]

Fig 1

Figure 1A		LDH measurement							
		0h	1h	2h	4h	8h	24h	48h	72h
SH	Test 1	-12.3	0.2	23.2	99.2	402.4	1697.2	4511.3	5945.1
	Test 2	2.1	3.1	50.9	101.3	775.1	1450.4	3099.5	5502.3
	Test 3	1.5	3.0	45.1	124.7	856.0	1749.6	3501.2	6200.2
	Test 4	39.7	57.0	139.6	219.3	1392.6	3050.0	6301.2	11845.6
	Test 5	1.2	2.5	24.5	94.4	795.7	1675.3	3311.2	6600.1
	Test 6	0.7	1.6	51.2	99.6	799.5	2050.3	4078.4	8199.2
SH + 1/8 HTK	Test 1	0.2	1.2	5.8	98.1	310.2	1300.4	2601.0	5980.1
	Test 2	2.0	16.1	24.2	110.2	356.4	699.6	1995.7	3994.1
	Test 3	1.3	13.2	20.1	142.5	563.0	826.0	2251.4	4012.3
	Test 4	1.0	10.0	14.9	99.8	650.2	1211.3	2479.0	4498.7
	Test 5	0.1	1.5	32.2	111.8	850.3	1289.7	3512.0	6990.2
	Test 6	33.3	90.2	125.3	240.1	1200.4	1551.1	3506.2	8012.4
SH + ¼ HTK	Test 1	1.0	2.2	3.6	49.2	31.2	801.0	1599.2	3998.9
	Test 2	2.0	11.2	4.6	99.5	59.4	749.5	1711.0	3092.1
	Test 3	1.6	3.7	2.1	141.1	129.4	653.2	1250.3	2999.7
	Test 4	7.6	5.6	18.5	99.3	128.4	802.4	1501.4	2845.0
	Test 5	1.0	3.5	15.0	60.1	129.7	752.3	2306.9	2394.5
	Test 6	30.1	87.3	33.1	170.5	1149.2	1294.8	2298.6	5295.0
SH + ½ HTK	Test 1	0.5	1.4	1.9	9.2	59.3	249.5	1394.5	2998.1
	Test 2	1.6	10.2	7.5	59.4	62.3	95.9	1699.8	3031.5
	Test 3	1.6	5.0	1.9	91.3	98.5	399.5	1199.7	4012.0
	Test 4	1.0	5.1	4.2	51.3	52.1	499.2	1011.2	2056.1
	Test 5	0.4	1.5	1.9	59.4	101.2	623.1	2031.1	2998.7
	Test 6	32.4	65.2	51.2	211.4	701.0	1150.2	2098.2	4059.1

Fig 1

Figure 1C		HIF-1a (ratio of DU)							
		0h	1h	2h	4h	8h	24h	48h	72h
Normoxia	Test 1	0.14	0.15	0.17	0.18	0.17	0.16	0.16	0.16
	Test 2	0.18	0.20	0.22	0.23	0.22	0.21	0.21	0.20
	Test 3	0.04	0.08	0.08	0.06	0.08	0.08	0.08	0.09
	Test 4	0.06	0.09	0.11	0.12	0.10	0.13	0.11	0.12
	Test 5	0.12	0.16	0.18	0.19	0.16	0.14	0.18	0.18
	Test 6	0.32	0.45	0.49	0.53	0.53	0.54	0.49	0.48
SH	Test 1	0.18	0.62	0.53	0.25	0.06	0.05	0.04	0.05
	Test 2	0.22	0.66	0.57	0.29	0.07	0.07	0.08	0.08
	Test 3	0.06	0.30	0.21	0.06	0.05	0.06	0.07	0.06
	Test 4	0.12	0.52	0.51	0.12	0.05	0.05	0.05	0.05
	Test 5	0.19	0.63	0.54	0.26	0.06	0.08	0.07	0.07
	Test 6	0.52	1.19	1.00	0.70	0.13	0.12	0.14	0.12
SH + ½ HTK	Test 1	0.15	0.56	0.64	0.60	0.24	0.25	0.29	0.31
	Test 2	0.25	0.76	0.84	0.86	0.34	0.38	0.50	0.51
	Test 3	0.05	0.34	0.35	0.34	0.07	0.08	0.18	0.10
	Test 4	0.06	0.20	0.24	0.23	0.08	0.07	0.17	0.18
	Test 5	0.12	0.23	0.23	0.18	0.29	0.30	0.35	0.36
	Test 6	0.33	0.62	0.66	0.69	0.46	0.48	0.69	0.78

Fig 1

Figure 1B		MTT assay							
		0h	1h	2h	4h	8h	24h	48h	72h
Normoxia	Test 1	100.1	102.0	105.0	100.0	101.0	100.0	101.0	100.0
	Test 2	90.0	95.1	98.0	103.0	90.0	94.0	95.0	90.0
	Test 3	98.5	99.0	100.0	98.0	94.0	93.0	94.0	95.0
	Test 4	114.6	115.1	109.0	100.0	102.0	101.0	102.0	103.0
	Test 5	85.0	91.5	92.0	93.0	86.0	85.0	85.0	86.0
	Test 6	112.0	112.1	120.0	115.0	129.0	122.0	124.0	132.0
SH	Test 1	102.0	110.0	106.0	105.0	105.0	84.0	69.0	66.0
	Test 2	100.0	99.0	97.0	95.0	88.0	64.0	44.0	32.0
	Test 3	93.0	90.0	82.0	80.0	72.0	45.0	20.0	4.0
	Test 4	103.0	102.0	98.0	88.0	72.0	48.0	28.0	20.0
	Test 5	90.0	82.0	80.0	75.0	70.0	46.0	26.0	18.0
	Test 6	118.0	117.0	113.0	109.0	100.0	76.0	56.0	48.0
SH + ½ HTK	Test 1	109.0	108.0	101.0	99.0	95.0	90.0	83.0	86.0
	Test 2	106.0	105.0	113.0	111.0	113.0	103.0	93.0	92.0
	Test 3	95.0	97.0	92.0	82.0	78.0	60.0	50.0	48.0
	Test 4	100.0	100.0	105.0	99.0	95.0	85.0	75.0	73.0
	Test 5	90.0	92.0	90.0	80.0	70.0	63.0	50.0	42.0
	Test 6	100.0	98.0	99.0	93.0	89.0	79.0	69.0	67.0

Fig 1

Figure 1D left panel		Caspase-3 (ratio of DU)			
		8h	24h	48h	72h
Normoxia	Test 1	0.15	0.17	0.16	0.15
	Test 2	0.18	0.20	0.17	0.16
	Test 3	0.10	0.08	0.11	0.10
	Test 4	0.56	0.62	0.56	0.52
	Test 5	0.05	0.07	0.06	0.06
	Test 6	0.18	0.20	0.20	0.18
SH	Test 1	0.45	0.55	0.75	0.80
	Test 2	0.50	0.60	0.90	1.20
	Test 3	0.40	0.50	0.70	0.90
	Test 4	1.50	1.65	1.90	2.60
	Test 5	0.20	0.25	0.45	0.70
	Test 6	0.60	0.70	0.90	1.10

Figure 1D right panel		pase-3 (ratio of DU)
		72 h
SH	Test 1	2.80
	Test 2	0.80
	Test 3	1.20
	Test 4	0.80
	Test 5	1.30
	Test 6	1.10
SH + 1/8 HTK	Test 1	2.00
	Test 2	0.70
	Test 3	0.80
	Test 4	0.60
	Test 5	1.00
	Test 6	0.80
SH + ¼ HTK	Test 1	1.50
	Test 2	0.40
	Test 3	0.50
	Test 4	0.30
	Test 5	0.60
	Test 6	0.50
SH + ½ HTK	Test 1	0.44
	Test 2	0.06
	Test 3	0.16
	Test 4	0.05
	Test 5	0.03
	Test 6	0.03

Fig 2

Figure 2A		H2O2 measurement (µM)								
		0h	1h	2h	4h	8h	24h	48h	72h	
Normoxia	Test 1	0.10	0.12	0.11	0.13	0.15	0.75	1.45	1.35	
	Test 2	0.01	0.02	0.03	0.02	0.08	0.24	0.36	0.12	
	Test 3	0.10	0.30	0.11	0.14	0.26	0.58	0.78	0.68	
	Test 4	0.01	0.02	0.01	0.02	0.04	0.12	0.33	0.23	
	Test 5	0.26	0.12	0.27	0.46	0.88	2.65	2.80	2.70	
	Test 6	0.06	0.08	0.07	0.02	0.10	0.30	0.25	0.30	
SH	Test 1	0.06	0.05	0.40	1.20	2.40	5.30	4.50	3.90	
	Test 2	0.01	0.03	0.10	1.90	3.80	4.70	3.90	3.20	
	Test 3	0.07	0.08	0.50	1.50	4.80	6.80	5.90	4.60	
	Test 4	0.04	0.05	0.30	1.10	3.20	3.70	3.10	2.60	
	Test 5	0.21	0.22	1.60	4.10	6.90	9.10	8.30	7.30	
	Test 6	0.02	0.03	0.20	1.30	2.60	4.60	3.80	2.60	
SH + ¼ HTK	Test 1	0.23	0.12	0.21	0.59	1.79	3.80	3.50	2.90	
	Test 2	0.02	0.03	0.02	0.06	0.58	1.78	2.00	1.80	
	Test 3	0.06	0.04	0.06	0.18	0.54	2.54	2.20	1.80	
	Test 4	0.03	0.02	0.03	0.09	0.47	2.47	2.20	1.60	
	Test 5	0.30	0.35	0.35	1.40	3.30	6.10	5.50	4.40	
	Test 6	0.02	0.01	0.03	0.09	0.57	2.57	2.2	1.5	

Fig 2

Figure 2C		LDH measurement							
		0h	1h	2h	4h	8h	24h	48h	72h
Normoxia	Test 1	1.200	1.400	16.756	29.057	65.980	170.100	365.200	804.248
	Test 2	1.941	2.100	9.900	19.100	29.640	57.980	99.210	399.200
	Test 3	-10.000	1.200	2.500	4.900	10.120	39.210	44.210	590.700
	Test 4	3.011	10.900	51.200	75.210	175.100	350.000	1420.000	2200.700
	Test 5	9.900	1.900	7.900	15.100	44.950	88.600	119.240	339.700
	Test 6	0.987	2.900	10.700	32.040	65.120	129.700	149.670	500.700
SH	Test 1	1.200	16.000	65.120	144.100	838.000	1940.000	4169.298	7336.000
	Test 2	10.300	45.100	175.600	349.500	1450.000	3001.000	6565.000	12211.000
	Test 3	7.840	14.970	73.200	149.200	801.000	1650.000	3511.000	6998.000
	Test 4	-15.100	1.200	8.110	68.100	561.000	1301.000	3489.000	4002.000
	Test 5	1.300	8.100	31.670	62.400	833.000	2099.000	3812.000	5997.000
	Test 6	2.100	10.120	41.230	83.100	559.000	1699.000	3496.000	7498.000
SH + HTK	Test 1	1.650	5.569	5.990	21.100	169.000	468.000	1282.974	2994.000
	Test 2	0.290	1.533	1.560	1.987	6.100	318.000	1594.000	3051.000
	Test 3	25.210	27.190	44.100	331.670	749.630	1198.000	2011.000	4568.000
	Test 4	-19.400	-4.100	0.200	1.856	56.100	240.000	999.800	3068.000
	Test 5	0.600	1.200	1.210	2.947	8.940	329.500	1002.000	2094.000
	Test 6	1.220	1.994	1.967	9.670	19.670	259.400	902.000	2130.000
H + HTK + 4008	Test 1	1.700	22.200	37.200	141.300	653.100	1146.100	2720.512	5568.129
	Test 2	-9.300	1.110	2.100	15.900	252.000	699.200	2294.000	4921.000
	Test 3	6.000	12.100	16.900	50.300	603.000	1100.100	3192.000	5981.000
	Test 4	1.200	4.900	9.800	29.700	406.000	800.200	1610.000	3451.000
	Test 5	10.500	74.100	121.100	550.100	1507.000	2199.000	4501.000	9421.000
	Test 6	0.200	19.210	39.400	49.500	503.000	998.700	1999.900	3851.000

Fig 2

Figure 2B		Ratio of DU			Figure 2D	NOX4 (ratio of DU)		
		NOX2	NOX4		Normoxia	Test 1	0.3200	
Normoxia	Test 1	0.89	0.45			Test 2	0.6900	
	Test 2	1.44	0.80			Test 3	0.1500	
	Test 3	0.56	0.20			Test 4	0.3300	
	Test 4	0.22	0.10			Test 5	0.1200	
	Test 5	1.20	0.60			Test 6	0.3200	
	Test 6	0.99	0.50		SH	Test 1	1.1107	
SH	Test 1	0.90	1.10			Test 2	0.9000	
	Test 2	2.10	1.50			Test 3	0.8000	
	Test 3	0.96	0.70			Test 4	1.8000	
	Test 4	0.62	0.80			Test 5	0.9000	
	Test 5	1.60	1.50			Test 6	1.1200	
	Test 6	1.30	1.00		SH + HTK	Test 1	0.3500	
SH + ¼ HTK	Test 1	1.10	1.05			Test 2	0.1200	
	Test 2	1.50	1.10			Test 3	0.5500	
	Test 3	0.80	0.50			Test 4	0.3300	
	Test 4	0.50	0.60			Test 5	0.6500	
	Test 5	1.50	0.90			Test 6	0.1500	
	Test 6	1.20	0.80		H + HTK + 4008	Test 1	1.4000	
SH + ½ HTK	Test 1	1.05	0.80			Test 2	1.3000	
	Test 2	1.45	1.00			Test 3	0.8000	
	Test 3	0.90	0.30			Test 4	1.8000	
	Test 4	0.52	0.40			Test 5	1.1000	
	Test 5	1.45	0.70			Test 6	1.2000	
	Test 6	1.25	0.60					

Fig 3

Figure 3A		LDH measurement							
		0h	1h	2h	4h	8h	24h	48h	72h
PBS	Test 1	2.000	3.142	9.561	15.235	43.976	98.027	348.351	869.298
	Test 2	1.500	2.112	4.987	2.114	24.697	64.578	230.114	361.000
	Test 3	14.098	15.978	41.235	15.380	77.640	112.100	551.230	1599.800
	Test 4	4.987	5.947	10.297	5.921	14.235	79.690	239.670	601.200
	Test 5	5.941	9.987	19.670	62.370	94.587	169.870	515.870	1401.000
	Test 6	-15.987	-19.789	-22.111	-9.940	4.678	64.970	195.278	401.000
100 µM H2O2	Test 1	1.667	13.707	50.340	83.118	250.011	631.120	939.033	1345.181
	Test 2	-21.240	-4.850	29.941	60.110	150.014	450.169	589.640	801.120
	Test 3	4.210	7.890	22.978	51.230	69.789	799.859	1198.700	1699.570
	Test 4	6.387	12.697	70.110	128.990	239.700	549.870	721.060	1197.560
	Test 5	1.500	2.145	23.145	49.879	99.854	271.120	749.870	1002.450
	Test 6	16.500	49.237	106.978	129.785	689.210	1102.670	1398.650	2005.978
400µM H2O2	Test 1	3.001	15.485	78.153	110.513	333.275	836.176	1215.430	1721.325
	Test 2	-18.110	-11.231	48.410	60.124	101.240	399.450	750.120	1001.360
	Test 3	11.987	46.912	125.120	179.684	430.120	799.850	1050.630	1997.640
	Test 4	6.120	19.254	99.854	139.456	581.114	1799.420	2397.400	3102.450
	Test 5	6.978	9.987	68.124	91.234	151.643	499.520	850.440	999.980
	Test 6	7.984	14.632	51.234	82.154	399.780	689.540	1050.630	1498.560
800µM H2O2	Test 1	3.500	33.223	86.368	131.465	430.370	1049.697	1338.858	1791.049
	Test 2	-15.987	-2.156	61.234	80.120	161.234	500.123	799.580	955.230
	Test 3	9.500	59.637	99.854	149.550	201.654	698.650	901.450	1120.450
	Test 4	14.957	79.854	151.789	190.240	599.789	1504.687	1908.210	2500.360
	Test 5	4.001	9.786	69.387	160.000	899.645	2004.697	2994.500	3498.670
	Test 6	5.005	19.790	49.678	79.946	299.789	506.974	799.560	911.250

Fig 3

Figure 3C		MTT assay							
		0h	1h	2h	4h	8h	24h	48h	72h
PBS	Test 1	100	102	101	101	100	100	100	101
	Test 2	89	90	91	92	92	92	87	91
	Test 3	105	100	103	103	103	91	102	104
	Test 4	100	115	117	117	115	116	122	125
	Test 5	115	90	88	87	89	87	87	85
	Test 6	90	103	103	102	102	115	103	102
400 µM H2O2	Test 1	101	100	96	92	85	60	50	42
	Test 2	88	85	81	77	75	42	30	22
	Test 3	106	112	108	104	97	70	60	55
	Test 4	99	92	84	80	75	60	50	40
	Test 5	90	93	87	83	69	45	33	20
	Test 6	116	120	120	116	109	88	78	73
00 uM H2O2 + 1/8 HT	Test 1	100	100	94	94	90	75	60	49
	Test 2	99	99	93	95	92	77	62	52
	Test 3	90	86	76	75	74	59	42	33
	Test 4	116	120	118	116	109	93	81	71
	Test 5	88	85	79	79	75	60	40	25
	Test 6	106	111	105	105	101	86	76	66
400 uM H2O2 + ¼ HTK	Test 1	100	100	96	92	89	80	70	68
	Test 2	87	88	84	80	75	67	55	53
	Test 3	107	106	102	98	93	86	76	75
	Test 4	100	100	97	92	88	80	70	68
	Test 5	91	90	86	82	80	70	60	55
	Test 6	115	116	112	108	103	97	89	88
400 uM H2O2 + ½ HTK	Test 1	100	99	98	98	93	85	76	72
	Test 2	115	114	111	113	114	103	93	87
	Test 3	88	86	87	85	75	70	62	60
	Test 4	108	107	107	107	100	92	83	79
	Test 5	99	98	97	97	90	82	73	69
	Test 6	91	90	89	89	86	78	69	65

Fig 3

	100 µM H2O2		LDH measurement							
			0h	1h	2h	4h	8h	24h	48h	72h
	H2O2 (no HTK	Test 1	3.216	23.707	50.340	93.118	450.011	731.120	939.033	1345.181
		Test 2	4.500	2.450	7.946	49.967	149.896	410.230	559.680	798.250
		Test 3	-14.110	1.500	15.974	59.875	239.674	401.260	745.630	1109.450
		Test 4	4.987	59.697	99.789	129.630	649.870	899.850	1101.230	1605.230
		Test 5	5.941	36.000	82.645	141.236	849.766	1051.230	1298.450	2099.780
		Test 6	15.000	19.941	48.967	91.258	401.210	902.300	999.563	1308.640
	+ 1/8 HTK	Test 1	2.300	5.108	13.515	52.135	174.493	471.401	916.205	1203.379
		Test 2	1.642	1.999	6.123	31.210	99.560	261.300	599.630	799.210
		Test 3	0.597	1.002	3.697	45.850	124.650	455.200	899.560	1150.000
		Test 4	2.100	5.012	14.562	52.146	199.564	595.630	1101.230	1411.000
		Test 5	7.450	16.987	38.945	100.560	349.580	699.210	1204.650	1726.300
		Test 6	0.510	0.612	3.984	30.658	101.230	351.240	799.210	895.620
	+ ¼ HTK	Test 1	3.210	8.065	25.098	43.152	127.031	398.471	581.918	922.432
		Test 2	1.800	4.954	19.560	34.980	89.650	301.230	471.200	799.210
		Test 3	0.650	2.120	18.236	36.110	99.560	395.640	578.260	899.640
		Test 4	1.950	2.995	19.560	39.120	119.465	398.500	498.650	901.230
		Test 5	11.200	28.645	52.697	80.120	228.450	501.230	754.630	1271.200
		Test 6	0.480	1.965	15.697	31.260	100.110	394.620	600.230	750.300
	+ ½ HTK	Test 1	2.667	3.819	15.489	24.417	93.149	236.307	560.176	810.274
		Test 2	6.297	3.124	22.100	31.210	107.220	250.340	600.198	899.561
		Test 3	1.499	2.891	4.987	7.854	60.397	199.670	498.239	650.270
		Test 4	14.112	9.453	29.945	44.963	129.634	301.160	681.684	1060.380
		Test 5	-13.000	0.112	2.900	6.129	58.011	202.360	521.967	799.456
		Test 6	4.169	3.924	11.967	32.897	110.236	229.463	499.876	649.520

Figure 3D		Caspase-3 (ratio of DU)								
	100uM	Test 1	1.780				Test 1	1.050		
		Test 2	1.901				Test 2	0.420		
		Test 3	1.492			+ 1/8 HTK	Test 3	0.510		
		Test 4	2.710				Test 4	2.430		
		Test 5	0.799				Test 5	0.970		
		Test 6	1.994				Test 6	1.130		
	400uM	Test 1	1.930				Test 1	0.480		
		Test 2	1.990				Test 2	0.311		
H2O2		Test 3	1.520		400 uM H2O2	+ 1/4 HTK	Test 3	0.230		
		Test 4	3.350				Test 4	1.264		
		Test 5	0.810				Test 5	0.442		
		Test 6	2.040				Test 6	0.315		
	800uM	Test 1	2.650				Test 1	0.190		
		Test 2	2.495				Test 2	0.230		
		Test 3	2.103			+ 1/2 HTK	Test 3	0.054		
		Test 4	4.297				Test 4	0.540		
		Test 5	1.789				Test 5	0.050		
		Test 6	2.946				Test 6	0.090		

Fig 3

400 µM H_2O_2		LDH measurement							
		0h	1h	2h	4h	8h	24h	48h	72h
H2O2 (no HTK	Test 1	2.364	39.485	81.153	131.513	533.275	936.176	1215.430	1721.325
	Test 2	5.110	59.600	119.600	198.210	800.200	1602.300	1880.000	2700.250
	Test 3	-9.987	8.200	39.456	81.220	369.855	711.690	1002.360	1498.560
	Test 4	0.511	29.700	60.127	99.687	250.167	308.640	499.870	801.560
	Test 5	5.967	49.857	89.967	131.690	549.780	655.690	899.670	1100.230
	Test 6	9.897	51.239	99.138	139.670	699.368	1411.690	1789.690	2499.859
+ 1/8 HTK	Test 1	3.165	16.908	36.047	80.857	115.739	269.665	940.075	1006.491
	Test 2	7.986	39.112	69.000	113.000	147.680	301.220	971.680	1637.020
	Test 3	5.001	31.690	49.987	95.190	130.219	284.191	954.190	1020.330
	Test 4	0.110	0.456	9.568	34.250	69.124	173.450	743.250	809.450
	Test 5	0.200	1.100	25.100	69.000	104.100	258.320	928.000	994.230
	Test 6	2.500	10.900	30.670	93.999	125.000	329.000	1110.000	1776.360
+ ¼ HTK	Test 1	3.985	14.919	30.373	58.818	121.768	218.481	831.906	1142.172
	Test 2	1.800	3.001	9.568	23.900	79.100	73.450	590.000	917.600
	Test 3	1.500	1.999	15.000	20.100	104.100	108.302	828.441	987.650
	Test 4	2.300	4.200	25.000	83.000	125.120	179.690	1020.520	1345.200
	Test 5	9.400	37.000	65.000	69.500	167.268	601.250	870.210	1428.100
	Test 6	4.950	28.000	39.670	96.400	130.265	134.911	850.960	1027.500
+ ½ HTK	Test 1	3.000	4.200	14.560	75.000	69.870	99.670	750.000	1145.200
	Test 2	9.400	10.000	40.000	59.870	117.000	280.000	650.000	928.100
	Test 3	4.950	13.000	31.690	86.000	100.210	134.911	630.120	1027.500
	Test 4	4.667	5.875	19.290	48.094	51.747	133.820	627.939	906.188
	Test 5	1.800	1.600	9.568	15.900	19.100	49.590	450.000	717.000
	Test 6	4.000	1.000	2.000	12.000	14.000	108.302	658.000	707.650

Fig 4B

	AUC of CL counts								
	PBS			HTK			Catalase		
	0 uM H2O2	1 uM H2O2	10 uM H2O2	0 uM H2O2	1 uM H2O2	1 0uM H2O2	0 uM H2O2	1 uM H2O2	10 uM H2O2
Test 1	11212	143385	894790	11200	110605	830755	10900	39021	375222
Test 2	12065	150661	900011	12001	110021	812140	15236	39621	389001
Test 3	17230	112140	891246	20111	90056	806324	10098	29013	205000
Test 4	11987	266354	1247410	9852	230465	1106402	6211	39611	509601
Test 5	7211	80012	686972	7203	55210	590001	17231	55621	150021
Test 6	9236	99124	766548	9001	89231	826211	7045	34697	552140

Fig 5

Figure 5A	CPR time				Figure 5B	Epinephrine dose (mg)			
	4'30"	4'30"+HTK	6'30"	6'30"+HTK		4'30"	4'30"+HTK	6'30"	6'30"+HTK
Rat number 1	0.6	0.2	0.5	9.5	Rat number 1	0.10	0.10	0.10	0.25
Rat number 2	0.2	1.8	14.5	8.5	Rat number 2	0.10	0.10	0.25	0.15
Rat number 3	0.2	3.2	2.5	3.5	Rat number 3	0.20	0.20	0.10	0.20
Rat number 4	3.2	1.0	3.5	3.5	Rat number 4	0.10	0.10	0.25	0.20
Rat number 5	0.5	0.3	13.5	14.0	Rat number 5	0.10	0.10	0.20	0.15
Rat number 6	3.2	0.2	3.5	2.0	Rat number 6	0.15	0.10	0.15	0.30
Rat number 7	0.3	0.2	0.5	1.0	Rat number 7	0.10	0.15	0.15	0.15
Rat number 8	1.0	3.0	2.0	3.0	Rat number 8	0.15	0.10	0.10	0.20
Rat number 9			1.0	0.5	Rat number 9			0.30	0.25
Rat number 10			2.5		Rat number 10			0.10	
Rat number 11			1.5		Rat number 11			0.20	
Rat number 12			3.0		Rat number 12			0.10	
Rat number 13			1.5		Rat number 13			0.30	
Rat number 14			1.0		Rat number 14			0.20	
Rat number 15			14.0		Rat number 15			0.30	

Fig 5

Figure 5D gical deficit (ND) scoring										
4'30''	Day 0	Day 1	Day 2	Day 3		4'30''+HTK				
Rat number 1	4.0	9.5	10.0	11.1		Rat number 1	4.3	8.5	9.4	10.0
Rat number 2	4.5	9.5	11.6	12.0		Rat number 2	5.0	9.8	12.0	12.0
Rat number 3	8.5	10.5	12.0	12.0		Rat number 3	4.5	9.1	10.0	11.2
Rat number 4	4.5	10.5	12.0	12.0		Rat number 4	4.3	8.8	10.1	11.4
Rat number 5	6.3	10.9	12.0	12.0		Rat number 5	6.0	10.1	11.4	12.0
Rat number 6	6.6	10.3	12.0	12.0						
6'30''						6'30''+HTK				
Rat number 1	1.3	3.6	5.1	6.5		Rat number 1	1.5	4.9	7.1	9.4
Rat number 2	1.1	2.9	4.0	6.2		Rat number 2	1.3	4.1	6.9	10.1
Rat number 3	1.5	3.4	5.6	7.8		Rat number 3	1.5	5.4	8.4	10.5
Rat number 4	1.1	2.4	5.4	7.9		Rat number 4	1.5	4.4	7.8	9.4
Rat number 5	1.3					Rat number 5	2.0	3.8		
						Rat number 6	1.5			

Fig 6

Figure 6A	H2O2 in blood (uM)				
	4'30"	4'30"+HTK	6'30"	6'30"+HTK	
Rat number 1	7.50	8.50	11.20	10.32	
Rat number 2	12.90	5.00	16.20	5.20	
Rat number 3	7.10	14.80	4.50	15.90	
Rat number 4	5.50	4.20	13.00	9.80	
Rat number 5	4.50	13.10			
Rat number 6		5.60			

Figure 6B	H2O2 in brain tissue (uM)			
	4'30"	4'30"+HTK	6'30"	6'30"+HTK
Rat number 1	3.25	2.91	7.13	3.85
Rat number 2	1.10	0.99	10.50	6.01
Rat number 3	5.80	5.70	5.51	3.94
Rat number 4	1.20	1.10	5.41	1.50
Rat number 5	4.90	4.99		
Rat number 6		1.91		

Figure 6C	NOX activity				
	4'30"	4'30"+HTK	6'30"	6'30"+HTK	
Rat number 1	2156	1985	3356	1613	
Rat number 2	2714	1995	4011	2105	
Rat number 3	1811	2809	2498	1310	
Rat number 4	1987	1397	3716	1467	
Rat number 5	2001	1708			
Rat number 6		2011			

Figure 6D	NOX4 mRNA			
	4'30"	4'30"+HTK	6'30"	6'30"+HTK
Rat number 1	0.90	1.70	1.56	0.59
Rat number 2	0.80	0.30	1.25	0.29
Rat number 3	1.30	0.90	2.20	0.51
Rat number 4	1.60	0.86	1.25	1.11
Rat number 5	0.40	1.10		
Rat number 6		0.30		
